# Massachusetts’ New Bureau

**Published:** 1902-03

**Authors:** 


### MASSACHUSETTS' NEW BUREAU.

IN a former issue we referred to the bill to abolish the Board of Cattle Commissioners of the "Bay State" and establish a "Bureau of Cattle" under the Commissioner of Agriculture. Whether it may prove a wise measure or otherwise, it is now on trial. The bill passed both branches of the Legislature, was signed by the Governor, and is now a State statute. From the profession's stand-point in that State one is at a loss to know whether it meets with its approval or disapproval. We pointed out one serious weakness in the bill, in that it did not fix this official chief as a veterinarian, though the control, restriction, and management of all animal diseases was to be under his direction. We trust this defect in the organic law will be remedied in the spirit of its enforcement, and that a veterinarian will be selected who will give earnest and painstaking care to more fully establish a better veterinary sanitary police control system throughout the State and place that Commonwealth among the foremost in our land in the management of all contagious and infectious diseases of lower animals.

